# Pelvic organ prolapse: a cross-sectional study during mass campaign in two hospitals in the city of Kananga in the Democratic Republic of Congo

**DOI:** 10.11604/pamj.2024.47.52.42470

**Published:** 2024-02-08

**Authors:** Antoine Tshimbundu Kayembe, Bertine Mayi Ilunga, John Mundende Muakuya, Andy Mbangama Muela, Rahma Raschid Tozin

**Affiliations:** 1 Department of Gynaecology and Obstetrics, Faculty of Medicine, University Notre-Dame of Kasayi, Kananga, Central Kasaï, Democratic Republic of Congo,; 2 Service of Gynecology, Saint Georges Hospital of Katoka, Kananga, Central Kasaï, Democratic Republic of Congo,; 3 Service of Gynecology, Bon-Berger Hospital of Tshikaji, Kananga, Central Kasaï, Democratic Republic of Congo,; 4 Department of Gynaecology and Obstetrics, Faculty of Medicine, University of Kinshasa, Kinshasa, Democratic Republic of Congo

**Keywords:** Epidemiology, clinical, therapeutic, pelvic organ prolapse, Kananga, Democratic Republic of Congo

## Abstract

**Introduction:**

pelvic organ prolapse is a dynamic pathology that can worsen or regress especially postpartum and is the basis of several disorders that bother the patient and alter her quality of life. This study aims to determine the epidemiological, clinical, and therapeutic profile of pelvic organ prolapse in the town of Kananga.

**Methods:**

this is a cross-sectional study of cases of pelvic organ prolapse recorded during the mass campaign organized in the Bon-Berger Hospital of Tshikaji and Saint Georges Hospital of Katoka in the town of Kananga, from January 1 to July 31, 2023. Non-probability convenience sampling was used to select cases.

**Results:**

we recorded 138 cases of prolapse out of 572 patients. The prevalence of pelvic organ prolapse is 24.12% with an average monthly incidence of 19.71 (SD: 4.23) cases per month. The prevalence of recurrence of pelvic organ prolapse is 8.69%. The average age of patients is 54.86 (SD: 11.36) years with an average parity of 7.62 (SD: 1.8) deliveries. Its preoperative symptomatology consists of the intravaginal mass associated with digestive and urinary disorders in 97.00% (n=130), stage III hysterocele predominates in 68.70% (n=92), surgical treatment is the most practiced in 91.79% (n=123) and hysterectomy associated with the treatment of cystocele and rectocele by vaginal surgical access is the most practiced in 80.60% (n=108).

**Conclusion:**

pelvic organ prolapse is a real public health problem in the city of Kananga, its symptoms are classic and its treatment is surgical via the vaginal route.

## Introduction

Pelvic organ prolapse is a reason for consultation and surgical interventions that are increasing in both gynecological and urological units [1,2]. Taking into account various factors, notably the increase in life expectancy, the number of patients suffering from genital prolapse will double in the coming decades [3]. Pelvic organ prolapse is diagnosed based on clinical elements and paraclinical examinations such as pelvic ultrasound, cysto-defecography, and Magnetic Resonance Imaging [4,5]. Its treatment can be medicinal or surgical [4,5]. Although pelvic organ prolapse is not fatal, it has a significant impact on the quality of life of patients and leads to serious social problems including loss of self-esteem [6-10].

The prevalence of pelvic organ prolapse varies from 2.9 to 97.7% worldwide depending on the study methods used. It is estimated from 2.9 to 11.4% when the method used is a symptom questionnaire [11-20] and from 31.8 to 97.7% when a clinical examination is carried out with the pelvic organs prolapse quantification (POPQ) [21-30]. In Asia and Africa, the prevalence of pelvic organ prolapse is not known due to a lack of surveys and studies in the general population [31]. In the Democratic Republic of Congo (D.R. Congo), the prevalence of pelvic organ prolapse is not known and there are no data yet to estimate its incidence to our knowledge. However, a hospital study carried out on this subject at Saint Joseph Hospital in Kinshasa found a frequency of 1.2% in 2019 [31]. The lack of epidemiological data on pelvic organ prolapse in our city justifies the present study, the objective of which is to determine the epidemiological, clinical, and therapeutic profile of pelvic organ prolapse in the city of Kananga in the D.R. Congo.

## Methods

**Study design and setting:** this is a cross-sectional study of a series of cases of pelvic organ prolapse recorded during the mass campaign organized in the gynecological departments of two General reference hospitals in the city of Kananga: Bon-Berger Hospital of Tshikaji and Saint Georges Hospital of Katoka, from January 1 to July 31, 2023. These two hospitals were chosen because of the presence of trained and experienced medical staff, the high attendance of patients suffering from pelvic organ prolapse, and the more or less free of pelvic organ prolapse through the various mass campaigns in the account of fistula cure. Therefore, these 2 hospitals constitute references for the treatment of pelvic organ prolapse in the city of Kananga.

**Study population:** we used the medical records of patients aged between 30 and 79 years suffering from pelvic organ prolapse and treated during the mass campaign in the gynecology departments of the Bon-Berger Hospital of Tshikaji and Saint Georges Hospital of Katoka in the town of Kananga in the Democratic Republic of Congo from January 1 to July 31, 2023. Our sampling is non-probabilistic for convenience. The sample size was determined by the limitation of our study in time and space. The following criteria allowed us to include the patients in the study: Patients aged between 30 and 79 years, suffering from pelvic organ prolapse, treated during the mass campaign in the gynecology departments of hospitals in the Bon-Berger Hospital of Tshikaji and Saint Georges Hospital of Katoka in the town of Kananga from January 1 to July 31, 2023, and whose medical file was complete. Patients who did not meet these inclusion criteria and had incomplete medical records were excluded.

**Collection of data:** the data was collected from registers of mass campaigns in gynecology departments, those of the operating room, medical files of patients in gynecology departments of two hospitals, and the data collection record. The variables of our study are clinical characteristics (age, parity, clinical symptoms, type, stage, and recurrence of pelvic organ prolapse) and therapeutic characteristics (type of treatment initiated, surgical approach, type of surgery, type of complications, and post-therapeutic evolution). The data collection was done as follows: we first identified the names of patients who had suffered from pelvic organ prolapse in the operating room and gynecology registers, then searched medical records based on the names of identified patients and finally transcribed the data from the medical records of these patients identified in the data collection sheet.

**Statistical analyzes:** data were analyzed using Statistical Package for Social Sciences (SPSS) software version 29. We used the average (SD) to present the quantitative variables and the proportion to present the qualitative variables.

**Ethical considerations:** our study was authorized by the Ethics Committee of the Kinshasa School of Health and by the Ethics Committee of these two hospitals in the city of Kananga. The reference number of the approval by the Ethics Committee is N°ESP/CE/19/2023. Principles of medical ethics and documentary studies rules have been respected: the data were collected confidentially and treated anonymously.

## Results

**Prevalence of pelvic organ prolapse and its recurrence:** during this mass campaign in the town of Kananga, we recorded 138 cases of pelvic organ prolapse among 572 patients in the gynecology departments of two hospitals, representing a prevalence of pelvic organ prolapse of 24.12% during the study period. Of these 138 cases of pelvic organ prolapse, 39 cases out of 245 patients were recorded in the gynecology department of the Bon-berger Hospital of Tshikaji, representing a prevalence of 15.91%, and 99 cases out of 327 patients in that of the Saint Georges Hospital of Katoka, a prevalence of 30.28%. Of these 138 cases of pelvic organ prolapse, 11 cases were recurrent prolapse, giving a recurrence prevalence of 8.69%. The average monthly incidence of pelvic organ prolapse cases is 19.71±4.23 cases per month. We recall that of these 138 cases of pelvic organ prolapse recorded, 134 available cases were included in the study and 4 were excluded because their files were not available.

**Clinical profile of pelvic organ prolapse:**the average age of patients suffering from pelvic organ prolapse was 54.86 (SD: 11.36) years with 89.60% of patients aged over 40 years. The average parity of our patients is 7.62 (SD: 1.8) deliveries with 97.30% of patients having a parity greater than or equal to 4 deliveries. The clinical profile consists of genital disorders associated with digestive and urinary disorders. Preoperative symptomatology consists mainly of the sensation of a pelvic or vaginal mass associated with dyspareunia and pelvic pain, stress urinary incontinence, urgent urination with or without urinary incontinence, and constipation. The most common type of pelvic organ prolapse is essentially hysterocele in 37.30% of cases. The most common stage of pelvic organ prolapse is stage III in 68.70% of cases (Table 1).

**Table 1:** clinical and therapeutical characteristics of pelvic organ prolapses

Age of patients	N=134	%
Below or equal to 40 years	14	10.4
Higher than 40 years	120	89.6
**Parity**		
Below or equal to 3	4	3.00
Greater or equal to 4	130	97.00
**Pre-operative symptom**		
Urinary incontinence of stress	25	18.70
Imperious micturition	12	9
Constipation	2	1.50
Anal incontinence	0	0.00
Dyspareunia et pelvic pain	95	70.90
**Stages of pelvic organ prolapses**		
Stage II	12	9
Stage III	92	68.70
Stage IV	30	22.40
**Type of pelvic organ prolapse**		
Cysto-colpocele (Anterior pelvic organ prolapses)	22	16.40
Hysterocele (middle pelvic organ prolapses)	50	37.30
Rectocele (posterior pelvic organ prolapse)	0	0.00
Association cystocele-hysterocele	33	24.60
Association hysterocele-rectocele	2	1.50
Association cystocele-hysterocele-rectocele	27	20.10
**Surgery**	123	91.79
Cystocele cure	22	16.4
Hysteropexia	4	3
Hysterectomy	68	50.7
Therapeutical association	40	29.90
**Vaginal surgical access**	84	62.24
**Complications**		
Intra operative	0	0.00
Postoperative	2	1.45

**Therapeutic profile:** surgical treatment was performed in 91.79% of cases and the vaginal approach in 62.24% of cases. Hysterectomy associated with anti-prolapse procedures is the most common type of treatment performed in 80.60% of cases. No intraoperative complications were recorded while infectious postoperative complications were the most frequent in 1.45% of cases. The post-therapeutic evolution was marked by the healing (or disappearance) of all cases of pelvic organ prolapse recorded, i.e. 100.0% (Table 1).

## Discussion

The objective of this study is to determine the epidemiological, clinical, and therapeutic profile of pelvic organ prolapse in the town of Kananga. The prevalence of pelvic organ prolapse is 24.12% with an average monthly incidence of 19.71 (SD: 4.23) cases per month. The prevalence of recurrence of pelvic organ prolapse is 8.69%. The average age of patients is 54.86 (SD: 11.36) years with an average parity of 7.62 (SD: 1.8) deliveries. Its preoperative symptomatology consists of the intravaginal mass associated with digestive and urinary disorders in 97.00% (n=130), stage III hysterocele predominates in 68.70% (n=92), surgical treatment is the most practiced in 91.79% (n=123) and hysterectomy associated with the treatment of cystocele and rectocele by vaginal surgical access is the most practiced in 80.60% (n=108). The prevalence of pelvic organ prolapse is 24.12% in the town of Kananga during the study period. Our prevalence is higher than those of Zhu *et al*. in China [32], Seven *et al*. in Türkiye [33], and Rodrigues *et al*. in Brazil [34] which are respectively 2.20%, 5.60% and 7.50%. It is lower than those of Wusu-Ansah in Ghana [35], Scherf *et al*. in Gambia [36], Megabiaw *et al*. in Ethiopia [37], Masenga *et al*. in Tanzania [38] which are 32.20%, 46.00%, 55.00%, and 64.60%. The prevalence of pelvic organ prolapse is higher in sub-Saharan Africa than in Western and Asian countries. The difference in prevalence can be explained by the comparatively high number of vaginal deliveries, difficult access to skilled deliveries, and the carrying of heavy loads or heavy physical work among Africans in sub-Saharan Africa as noted by Masenga *et al*. in Tanzania [38]. This is also the case in our environment.

In the study by Nygaard *et al*. in the USA, the prevalence of pelvic organ prolapse was 1.90% among African-American women, 2.80% among white women, and 5.10% among Hispanic women [18]. These Nygaard prevalences are much lower than ours. Masenga's observation described above [38] can also explain this difference in the prevalence of pelvic organ prolapse between African women residing in the USA and those residing in sub-Saharan Africa, specifically in our environment. The prevalence of pelvic organ prolapse is 15.91% at the Bon-berger Hospital in Tshikaji while it is 30.28% at the Saint George Hospital in Katoka. The difference in prevalence in these two hospitals can be explained by their geographical location: eccentric of the Bon-berger Hospital of Tshikaji, located 9-10 km from the city center of Kananga with more or less difficult access for patients from all over the city explaining its low prevalence while the situation is the opposite for the Saint Georges Hospital in Katoka, located 1-2 km from the city center with easy access for patients from the city of Kananga.

The prevalence of recurrence of pelvic organ prolapse is 8.69% in our town of Kananga, much lower than those of De Tayrac in France [39], Brandon *et al*. in the USA [40], by Smith *et al*. in Ireland [41] and Lucot *et al*. in England [42] which are more than 30.00%. Our low prevalence of recurrence can be explained by the good mastery of prolapse surgery techniques by practitioners in our city. Our prevalence of recurrence is higher than that of Kinshasa in the DRC [31] which was 2.00%. This difference in these prevalences of recurrence can be explained by the high average parity of 7.62±1.60 deliveries and the heavy physical work of women in the city of Kananga in the provinces compared to those in Kinshasa whose average parity was 5.37±0. 31 deliveries without heavy physical labor [31]. The monocentric nature of the Kinshasa study [31] may also explain its lower prevalence of recurrence than ours.

The most common complaint in gynecological consultation is genital associated with urinary and digestive disorders in 97.00% of cases and the preoperative symptoms are essentially vaginal mass in 97.00% of cases, stress urinary incontinence in 18.70% of cases, Urgent urination in 9.00% of cases, constipation in 1.50% of cases and dyspareunia in 70.90% of cases. Our results corroborate those reported by Rivaux *et al*. in France [43], by Tshimbundu *et al*. in Kinshasa [31]. These symptoms are linked on the one hand to the anatomical lesions of the anal levator and the pelvic fascias secondary to direct trauma and the abolition of innervation weakening the pelvic floor and on the other hand to the increase in intra-abdominal pressure with elevated intravesical pressure [44,45]

The middle pelvic organ prolapse consisting of hysterocele is the most common type of pelvic organ prolapse: isolated in 37.30% of cases, associated with anterior pelvic organ prolapse in 24.60% of cases, with posterior pelvic organ prolapse in 1.50% of cases and two preceding ones in 20.10% of cases. Our results are not consistent with those of Tshimbundu *et al*. [31], Blain *et al*. [29], Handa *et al*. [ 21]. Amblard *et al*. [46]. Our sequence of pelvic organ prolapse types consisting of hysterocele-cystocele-rectocele is different from other African studies which reported sequences consisting of cystocele-hysterocele-rectocele [31,47,48] and cystocele-rectocele-hysterocele [21,29,30]. This difference in sequences is only based on epidemiological observations.

As for the stages of pelvic organ prolapse, stage III is the most encountered with 68.70% of cases while stage I is not present. Our results corroborate those of Tshimbundu *et al*. [31], of Miaadi *et al*. [49], and Thomin *et al*. [50]. Handa reports that advanced stages (III and IV) of pelvic organ prolapse are associated with a poor prognosis due to their numerous symptoms which prompt patients to consult, whereas stage I is asymptomatic and requires no treatment [47]. The non-presence of stage I in our series of cases determines the symptomatic nature of pelvic organ prolapse in our city and can be explained by its regression towards stage 0 or its evolution towards stages II, III, and IV as reported by many authors in the literature [19,31,51].

Surgical treatment is more practiced in 91.79% of cases. Our results match those of Tshimbundu *et al*. in Kinshasa [31] and Boulanger *et al*. in France [52,53]. Villet and De Tayrac report that surgery in the treatment of pelvic organ prolapse is ideal for correcting anatomical lesions without causing new disorders [39,54]. This is the aim of the surgery performed in our city. No pessary treatment was recorded in our case series, although the rate of pessary use exceeds 11.00% in many studies [53,55,56]. This can be explained by the strategy of favoring surgical repair in all healthy patients in our environment, as Boulanger *et al*. reported [52,53]. Clemons *et al*. report that the increase in the number of externalized pelvic organ prolapses makes their control with pessaries very difficult and the long-term complications caused by the use of pessaries limit their use [57]. These two arguments also explain the non-use of pessaries in our series of cases.

The vaginal surgical approach is more used in 62.24% of cases. Our results are in agreement with those of Tshimbundu *et al*. [31] and De Tayrac *et al*. [39]. Boulanger and Maher report that the vaginal surgical approach has the advantages of reducing the operating time compared to that of abdominal surgery and allowing a more rapid return to activities [52,53,58]. These advantages explain our results. Laparoscopic surgical access requires laparoscopic equipment and medical personnel trained for its use. These two conditions are not yet met in our city's hospitals. This explains the non-use of this access in our series of cases. De Tayrac reports that the laparoscopic surgical approach does not allow the treatment of stress urinary incontinence and vulvar gaping [39]. This can also explain its non-use in our environment.

Hysterectomy associated with cystocele and rectocele treatment is the most common type of treatment performed in 80.60% of cases. Our results match those of the Kinshasa study [31] and can be explained by the predominance of pelvic organ prolapse of the middle type isolated and associated with the anterior and posterior type and by the average age of our patients is 54.86±11.36 years with 89.60% of patients aged over 40 years. Boulanger reports that hysterectomy performed in pelvic organ prolapse surgery must be associated with procedures to correct the prolapse, and uterine conservation is only carried out if there are no uterine anomalies discovered in the oncological cervicovaginal smear, systematic endometrial biopsy, and pelvic ultrasound [52,53]. Unfortunately, these systematic pre-operative examinations were not carried out in the hospitals of our city, due on the one hand to the ignorance of practitioners and on the other hand to the low social and economic level of our patients.

In the case series of Korahanis *et al*. in France, the installation of transvaginal polypropylene prostheses associated with the treatment of pelvic organ prolapse was the most practiced with more than 80.00% of cases [59]. This is not the case in our series of cases where the prosthesis use rate was 0%, due to the unavailability of these materials. No intraoperative complications were recorded (0.00%) in our case series while Tshimbundu *et al*. reported an intraoperative complication rate of 2.00% [31] and De Tayrac *et al*. an overall rate of intraoperative complications of 5.80%, after the use of two approaches [39]. The difference in complication rates can be explained by the mastery, despite the lack of equipment, of surgical techniques for the management of pelvic organ prolapse by practitioners in our field.

The Kinshasa study reports the absence of post-operative complications [31]. while in those of Lovatsis *et al*. and Hefni *et al*., postoperative complications were very rare, consisting mainly of lower back pain in 0.40-6.50% of cases [60,61]. These results are almost similar to ours where post-operative complications were present in 1.45% of cases in a mild infectious form. These results express the best quality of care in our city. The weakness of the study is the failure to evaluate genetic risk factors and not being involved in the occurrence of pelvic organ prolapse and its strength is to be the first to study the epidemiological, clinical, and therapeutic profile of pelvic organ prolapse in city hospitals of Kananga in the DRC.

## Conclusion

This study presents the real existence of pelvic organ prolapse in the city of Kananga. The prevalence of pelvic organ prolapse is 24.12% with its recurrences of 8.69%. Its preoperative symptomatology consists of the vaginal mass associated with digestive and urinary disorders, stage III hysterocele predominates, surgical treatment is the most practiced and hysterectomy associated with the treatment of cystocele and rectocele by surgical access vaginal is the most practiced. Our results can be used to raise scientists' awareness of the studies of pelvic organ prolapse and provide the basis for in-depth studies on pelvic organ prolapse to improve its treatment in the city of Kananga in the Democratic Republic of Congo.

### What is known about this topic

- *Pelvic organ prolapse is a dynamic disease that can worsen or recede above all in pregnant women during the postpartum period;*
- *It comprises a great recurrence risk after surgical treatment and it causes urinary, digestive, and genital problems that hamper the patients;*
- *The lack of epidemiological data on pelvic organ prolapse in hospitals in the city of Kananga in D. R. Congo*.

### What this study adds

- *The prevalence of pelvic organ prolapse is 24.12% with its recurrences at 8.69%;*
- *Its preoperative symptomatology consists of the vaginal mass associated with digestive and urinary disorders, stage III hysterocele predominates and is treated surgically by accessing the vaginal;*
- *Our results can be used to raise scientists' awareness of the studies of pelvic organ prolapse and serve as a basis for more in-depth studies on pelvic organ prolapse to improve its care in Kananga, D. R. Congo*.
